# Angiocentric Variant of Primary Cutaneous CD30-Positive Anaplastic Large Cell Lymphoma: A Case Report and Literature Review

**DOI:** 10.7759/cureus.109754

**Published:** 2026-05-27

**Authors:** Mariana Herrera Ocampo, Guillermo Roa Alvarez, Mario Shuchleib Cukiert, Marcela Hernández Vera, Laura S Parra Jaramillo, Maria E Vega Memije

**Affiliations:** 1 Department of Dermatology, Hospital General "Dr. Manuel Gea Gonzalez", Mexico City, MEX; 2 School of Medicine and Health Sciences, Tecnológico de Monterrey, Mexico City, MEX; 3 Department of Dermatopathology, Hospital General "Dr. Manuel Gea Gonzalez", Mexico City, MEX

**Keywords:** alk-negative lymphoma, angiocentric variant skin lymphoma, cd30 expression, cd30-positive lymphoproliferative disorders, chronic ulcer, cutaneous t-cell lymphoma, dermatopathology immunohistochemistry, primary cutaneous anaplastic large cell lymphoma

## Abstract

Anaplastic large cell lymphoma (ALCL) is part of the spectrum of CD30-positive lymphoproliferative disorders within cutaneous T-cell lymphomas. These disorders account for approximately 25%-30% of all primary cutaneous lymphomas (PCLs). Primary cutaneous ALCL (pcALCL) is an uncommon entity with heterogeneous clinical and histopathologic features, often posing a diagnostic challenge.

We report a 79-year-old man who presented with a three-month history of a non-healing ulcer on the right lower limb following minor trauma. The lesion measured 6.7 × 9.8 × 0.3 cm and showed irregular erythematoviolaceous borders with areas of eschar. Histopathologic evaluation revealed a dense infiltrate of large atypical mononuclear cells with pleomorphic nuclei, prominent nucleoli, and atypical mitoses, accompanied by angiocentric and angiodestructive features. Immunohistochemistry demonstrated CD30 positivity in more than 75% of atypical cells and negative anaplastic lymphoma kinase (ALK) expression, confirming pcALCL. The patient responded favorably to electron beam radiotherapy.

This case underscores the importance of early biopsy in atypical or treatment-refractory ulcers and highlights the diagnostic relevance of recognizing rare variants such as the angiocentric subtype.

## Introduction

Primary cutaneous lymphomas (PCLs) are extranodal non-Hodgkin lymphomas that are limited to the skin at the time of diagnosis, with no evidence of extracutaneous disease [1]. They represent a heterogeneous group of neoplasms with distinct clinical, histologic, immunophenotypic, and prognostic characteristics. For this reason, clinicopathologic correlation is essential. The 2018 WHO-EORTC classification organizes primary cutaneous lymphomas into major categories, including cutaneous T-cell lymphomas, cutaneous B-cell lymphomas, and primary cutaneous CD30-positive lymphoproliferative disorders [2].

Primary cutaneous CD30-positive lymphoproliferative disorders include lymphomatoid papulosis and primary cutaneous anaplastic large cell lymphoma. Primary cutaneous anaplastic large cell lymphoma (pcALCL) is one of the most frequent cutaneous T-cell lymphomas after mycosis fungoides and is defined by a dermal infiltrate composed of large atypical lymphoid cells with strong CD30 expression [3]. Although its prognosis is usually favorable, diagnosis may be delayed because its clinical appearance can resemble chronic infection, traumatic ulceration, pyoderma gangrenosum, or other inflammatory dermatoses. Rare histologic variants, such as the angiocentric pattern, increase this diagnostic difficulty because vascular invasion and destruction may suggest vasculitis, infection, or more aggressive lymphoid neoplasms. The angiocentric variant of pcALCL is particularly uncommon and diagnostically significant because its vascular invasion, necrosis, and hemorrhagic features may closely resemble vasculitic disorders, chronic infection, pyoderma gangrenosum, or extranodal NK/T-cell lymphoma. Angiocentric refers to the tendency of atypical lymphoid cells to surround and infiltrate vascular structures, occasionally causing vascular destruction. We report this case to emphasize the importance of early biopsy, clinicopathologic correlation, and systemic staging in chronic treatment-refractory ulcers with atypical features. This report includes a narrative discussion of the relevant literature regarding the clinicopathologic features, differential diagnosis, and management of pcALCL.

## Case presentation

**Figure 1:**
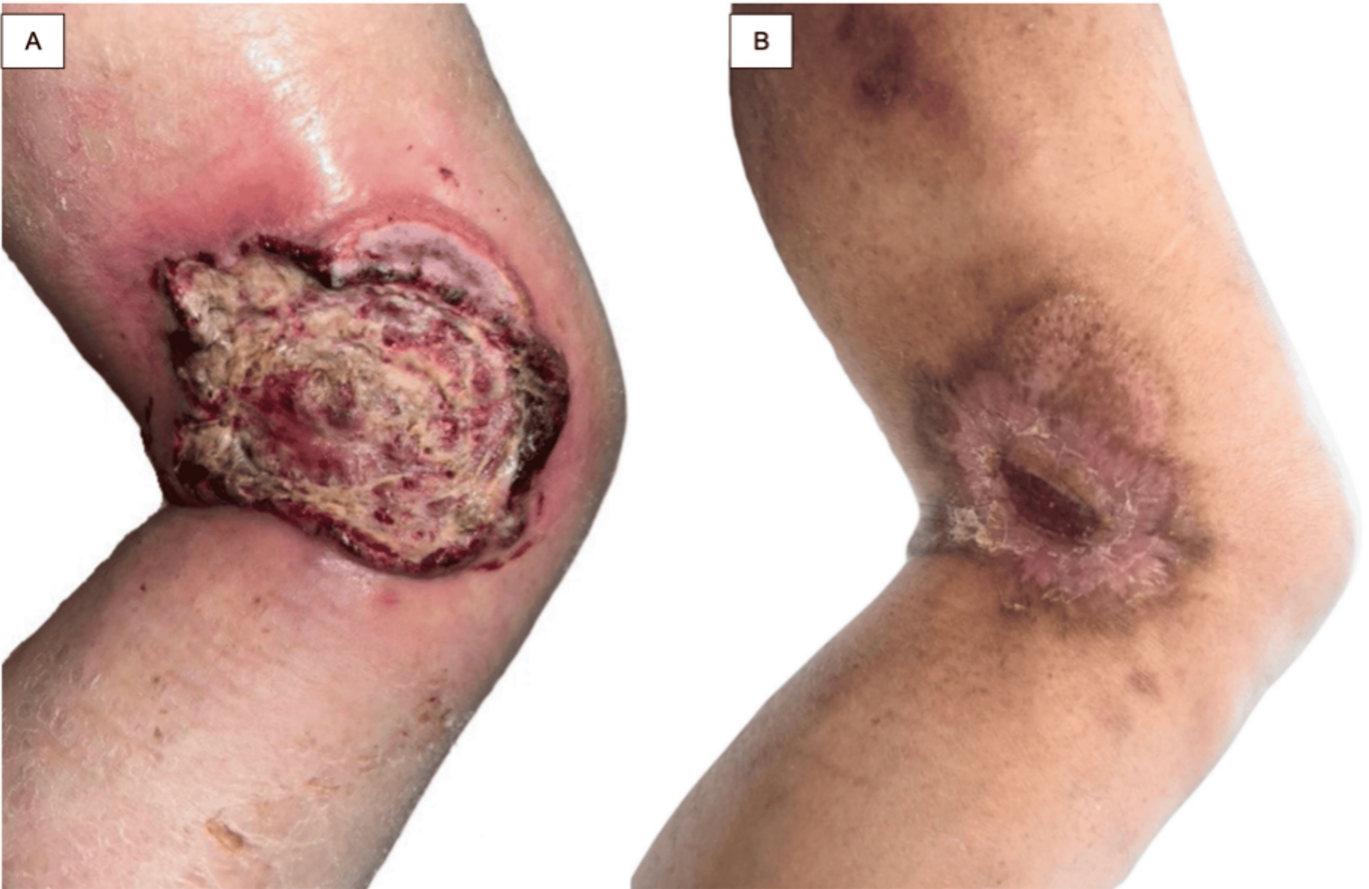
Clinical images A: Initial ulcer measuring 6.7 × 9.8 × 0.3 cm located on the lateral aspect of the distal third of the thigh. B: Residual dermatosis after five months of treatment with advanced dressings and electron beam radiotherapy, showing near-complete re-epithelialization.

A 79-year-old male patient presented to the wound care clinic of the Hospital General “Dr. Manuel Gea Gonzalez” with a dermatosis localized to the right lower limb, involving the lateral aspect of the distal third of the thigh. The lesion consisted of a 6.7 × 9.8 × 0.3 cm ulcer with irregular erythematoviolaceous borders and focal areas of eschar. The wound bed was composed of approximately 80% fibrin and 20% necrotic tissue (Figure 1A).

Table 1 presents the clinical timeline of the patient.

**Table 1:** Clinical timeline of the patient pcALCL: primary cutaneous anaplastic large cell lymphoma, CT: computed tomography, PET-CT: positron emission tomography-computed tomography

Time	Clinical event
3 months before consultation	Ulcer appeared after minor trauma
Following weeks	Multiple empiric antibiotic regimens without improvement
Initial wound care evaluation	Advanced dressings initiated
Subsequent evaluation	Incisional biopsy performed
Histopathologic diagnosis	pcALCL, angiocentric variant
Staging workup	CT and PET-CT performed
Treatment phase	Electron beam radiotherapy initiated
5-month follow-up	Near-complete re-epithelialization

The patient reported that the lesion had appeared three months before consultation after a fall from standing height. Since lesion onset, the patient had received multiple empiric oral antibiotic regimens prescribed prior to referral to our institution, without clinical improvement. Complete records regarding antibiotic agents and treatment duration were unavailable at the time of evaluation. He denied fever, night sweats, weight loss, or other systemic symptoms. The patient also denied diabetes mellitus, peripheral vascular disease, immunosuppression, chronic dermatologic disease, prior malignancy, HIV infection, or use of immunosuppressive medications. On physical examination, no palpable lymphadenopathy was identified. Given the history of trauma, the ulcerated morphology, and the lack of response to antibiotics, the initial diagnostic considerations included infected traumatic ulcer and pyoderma gangrenosum.

Cultures for bacteria, fungi, and mycobacteria were obtained and showed no growth of pathogenic microorganisms. Initial management consisted of advanced wound dressings. Because the ulcer persisted despite conservative treatment and had atypical clinical features, an incisional biopsy was performed. The histopathologic material was reviewed by dermatopathologists with experience in neoplastic disease, a dermatopathologist specialized in cutaneous lymphomas, and a hematopathologist.

**Figure 2:**
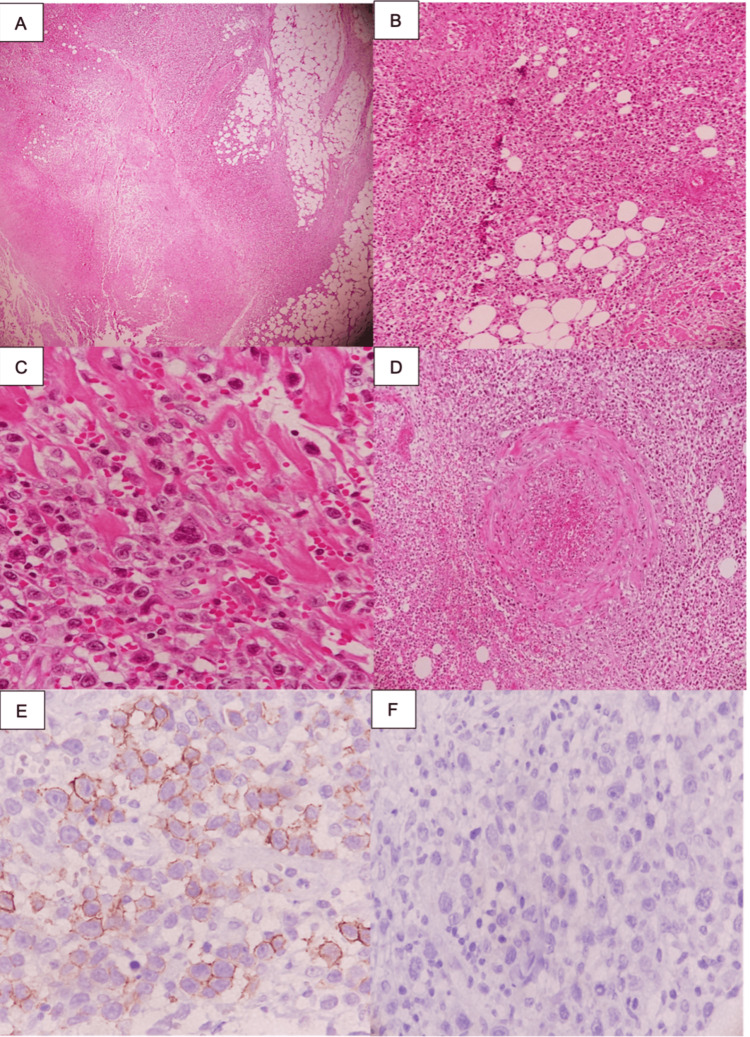
Histopathologic and immunohistochemical findings A: Dense infiltrate of large mononuclear cells extending into the subcutaneous tissue (hematoxylin and eosin, original magnification, 40×). B: Mononuclear cells dissecting collagen fibers (hematoxylin and eosin, original magnification, 100×). C: Infiltrate of large atypical mononuclear cells with irregular nuclei (hematoxylin and eosin, original magnification, 400×). D: Focal areas of necrosis and hemorrhage with vascular involvement (hematoxylin and eosin, original magnification, 100×). E: CD30 positivity in more than 75% of neoplastic cells (immunohistochemistry, original magnification, 400×). F: Negative ALK immunostaining (immunohistochemistry, original magnification, 400×). ALK: anaplastic lymphoma kinase

Routine hematoxylin and eosin staining showed a dense infiltrate involving the dermis and subcutaneous tissue. The infiltrate was composed of large atypical mononuclear cells with irregular nuclei, prominent nucleoli, scant cytoplasm, and atypical mitotic figures (Figure 2A-2C). Focal areas of necrosis and hemorrhage were also present. Importantly, there was evidence of vascular invasion and angiodestruction, with atypical cells arranged around and within vessel walls, consistent with an angiocentric growth pattern (Figure 2D). These angiocentric and angiodestructive findings were particularly relevant because they explained the ulcerative and necrotic clinical appearance and contributed to the initial clinical suspicion of an inflammatory or infectious process. These findings excluded the initial clinical suspicions and prompted immunohistochemical evaluation.

Immunohistochemistry was performed using CD3, CD4, CD8, CD30, and anaplastic lymphoma kinase (ALK). The atypical lymphoid cells showed partial positivity for CD3 and CD4, while CD8 expression was limited in the neoplastic infiltrate. CD30 was positive in more than 75% of atypical cells, confirming the diagnosis of a CD30-positive lymphoproliferative disorder (Figure 2E). ALK staining was negative (Figure 2F). Additional markers, including CD2, CD5, CD7, CD20, PAX5, EMA, cytotoxic markers, EBER, Ki-67, and T-cell receptor clonality studies, were not performed because of limited tissue availability and institutional resource limitations.

**Figure 3:**
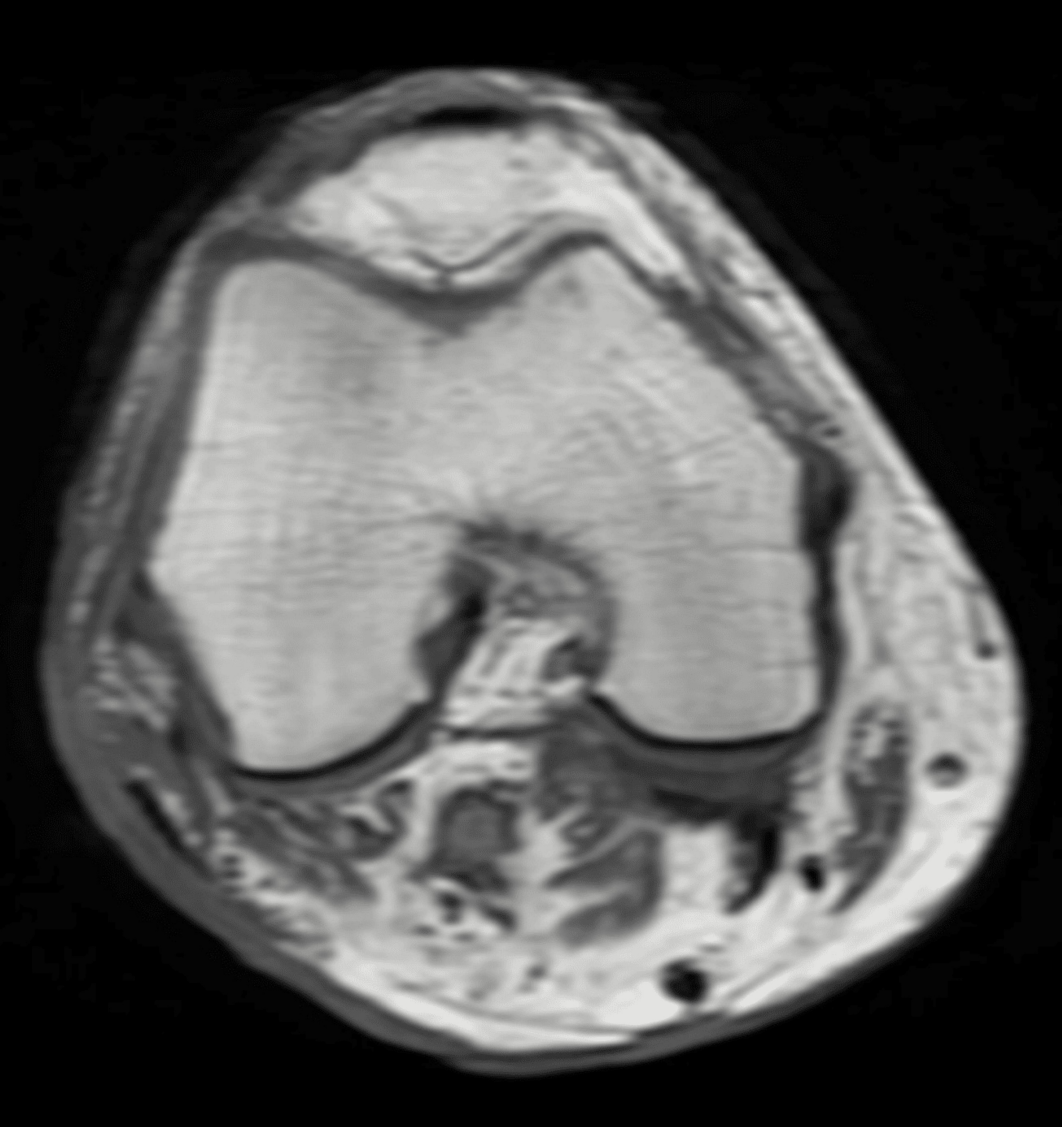
MRI findings of primary cutaneous anaplastic large cell lymphoma MRI of the right lower extremity demonstrating an ulcerated soft tissue lesion involving the lateral aspect of the distal thigh, with adjacent inflammatory changes and reactive-appearing regional lymphadenopathy. No evidence of deep muscular invasion or distant extracutaneous involvement was identified. MRI: magnetic resonance imaging

**Figure 4:**
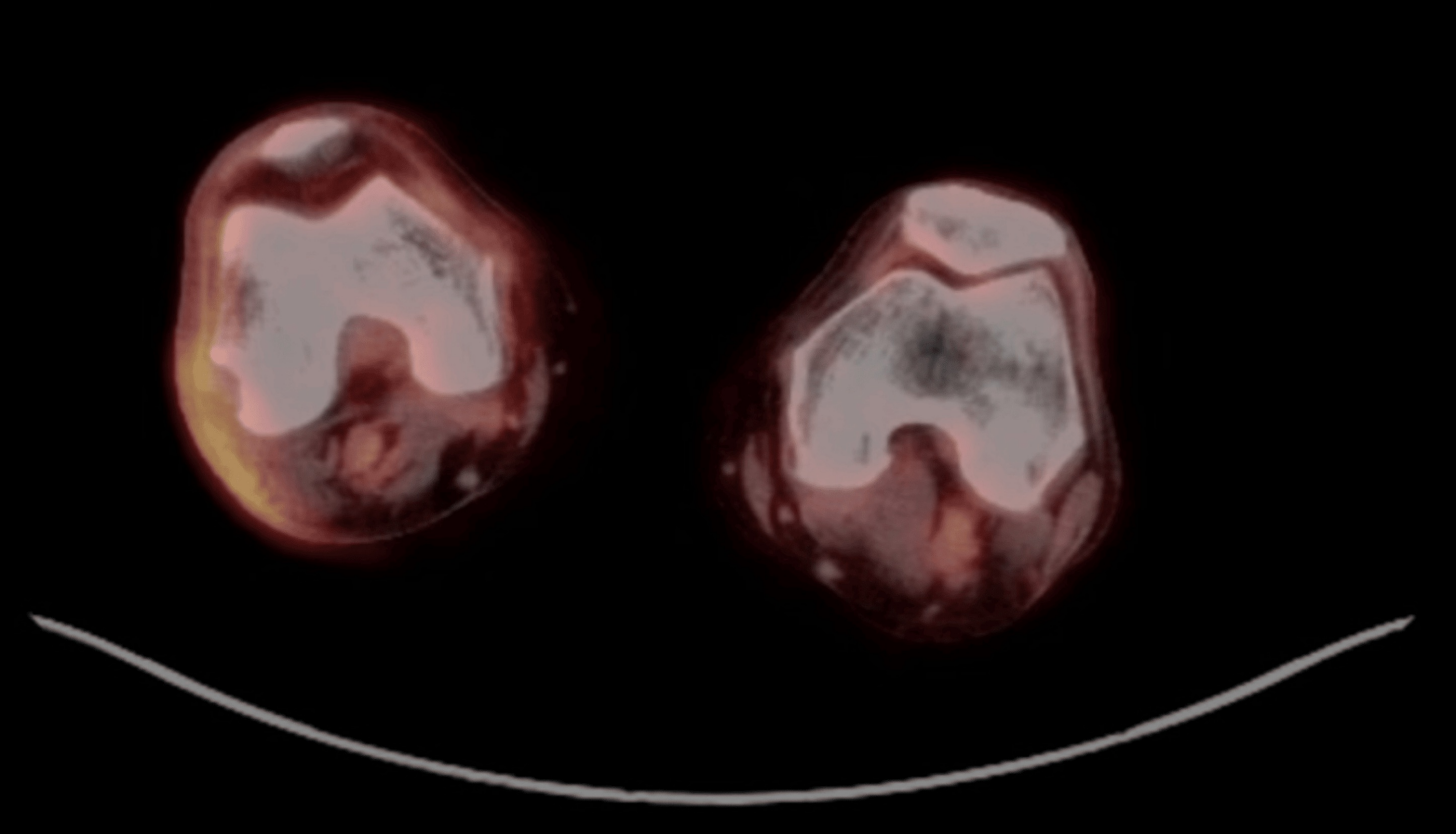
PET-CT findings 18F-FDG PET-CT demonstrating hypermetabolic uptake corresponding to the ulcerated cutaneous lesion of the right distal thigh, associated with ipsilateral popliteal and inguinal lymph nodes showing reactive metabolic activity without evidence of distant systemic disease. 18F-FDG PET-CT: 18-fluorodeoxyglucose positron emission tomography-computed tomography

As part of the staging workup, laboratory and imaging studies were requested to assess possible extracutaneous involvement. Laboratory results were within normal parameters. Computed tomography of the abdomen and right lower limb showed inflammatory-appearing lymph nodes in the popliteal region (Figure 3). Positron emission tomography with 18-fluorodeoxyglucose demonstrated an ulcerated cutaneous lesion in the right thigh, associated with ipsilateral popliteal and inguinal lymph nodes that were increased in size but had reactive-appearing lymphadenopathy without radiologic evidence of distant extracutaneous disease (Figure 4). No distant systemic involvement was documented. The patient underwent localized electron beam radiotherapy directed to the right distal thigh lesion. Treatment was completed over two therapeutic cycles with favorable tolerance and no significant acute adverse effects documented in the medical record. Follow-up evaluation at five months demonstrated marked reduction in ulcer size with near-complete re-epithelialization (Figure 1B). Complete dosimetric information and fractionation details were unavailable.

## Discussion

Anaplastic large cell lymphomas may be primary cutaneous or systemic. These entities share morphologic features but differ substantially in clinical behavior, prognosis, and treatment [3]. Primary cutaneous anaplastic large cell lymphoma is limited to the skin at presentation, whereas systemic anaplastic large cell lymphoma primarily involves lymph nodes and may secondarily affect the skin. Distinguishing between these entities is therefore mandatory, particularly because systemic disease requires a different staging and therapeutic approach.

The WHO classification incorporates genetic criteria that distinguish ALK-positive and ALK-negative anaplastic large cell lymphomas [4]. ALK-positive tumors commonly harbor the t(2;5)(p23;q35) translocation, leading to constitutive ALK kinase activation and oncogenic signaling. These cases are more frequent in children and young adults and are usually systemic. In contrast, primary cutaneous anaplastic large cell lymphoma is typically ALK-negative and occurs more often in older adults, which is consistent with the present case [3,4].

Primary cutaneous anaplastic large cell lymphoma accounts for a relevant proportion of CD30-positive cutaneous lymphoproliferative disorders and approximately 8% of primary cutaneous lymphomas [2,5]. It most commonly presents as a solitary or localized nodule, plaque, or tumor that may grow rapidly and ulcerate. Lesions frequently arise on the trunk, face, or extremities [6,7]. Most patients have localized disease, although a minority may develop multifocal lesions or extracutaneous spread, usually involving regional lymph nodes [6].

Histologically, the classic finding is a dense dermal infiltrate of large pleomorphic lymphoid cells, often described as “hallmark” cells, with irregular or horseshoe-shaped nuclei [8]. Several morphologic variants have been described, including neutrophilic-eosinophilic, epidermotropic, and angiocentric forms. The angiocentric variant is characterized by infiltration and destruction of small- and medium-sized vessels, often accompanied by fibrin deposition, necrosis, and hemorrhage [9]. This vascular pattern is especially relevant because it may simulate vasculitis, infection, extranodal NK/T-cell lymphoma, or ulcerative inflammatory dermatoses. This diagnostic overlap may delay histopathologic confirmation and expose patients to unnecessary antimicrobial or anti-inflammatory therapies before an underlying lymphoproliferative disorder is recognized.

Immunohistochemistry is central to diagnosis. Strong CD30 expression in more than 75% of atypical cells is required, although CD30 expression alone is not specific and must be interpreted with morphology and clinical findings [6]. T-cell markers such as CD2, CD3, CD4, CD5, CD7, and CD8 may show variable expression [10]. ALK negativity supports a primary cutaneous origin, while ALK positivity should raise concern for systemic anaplastic large cell lymphoma [10,11]. Molecular studies, particularly T-cell receptor gene rearrangement analysis, may be useful when clonality is uncertain [10,12]. DUSP22 rearrangements, TP63 abnormalities, and NPM1-TYK2 fusions have also been described in subsets of cases, although their prognostic and therapeutic implications continue to be investigated [6].

This report has several limitations. Complete information regarding prior antibiotic exposure and radiotherapy dosimetry was unavailable because part of the patient’s treatment occurred before referral to our institution. In addition, extended immunophenotypic and molecular studies, including Ki-67, EBER, EMA, cytotoxic markers, and T-cell receptor clonality analysis, could not be performed because of limited tissue availability and institutional resource limitations.

The differential diagnosis includes lymphomatoid papulosis, mycosis fungoides with large cell transformation, systemic anaplastic large cell lymphoma, pyoderma gangrenosum, and other ulcerative or angiodestructive processes [5,10]. Lymphomatoid papulosis usually presents with recurrent papules or nodules that undergo spontaneous regression, whereas primary cutaneous anaplastic large cell lymphoma tends to persist and enlarge. Mycosis fungoides with large cell transformation is suggested by a previous history of patches or plaques. Systemic anaplastic large cell lymphoma must be excluded through clinical evaluation, imaging, and immunophenotyping.

Localized primary cutaneous anaplastic large cell lymphoma is effectively treated with surgical excision or radiotherapy [13]. Electron beam therapy is frequently used in cutaneous lymphomas and offers good local control with acceptable toxicity [14]. Systemic therapy, including CHOP-based chemotherapy or targeted therapy with brentuximab vedotin, is reserved for multifocal, refractory, or extracutaneous disease [15]. Overall prognosis is favorable, with 10-year survival approaching 90% [2,6]. Nevertheless, lower extremity involvement, extensive disease, and extracutaneous spread have been associated with less favorable outcomes [6,16].

This case emphasizes the need to consider cutaneous lymphoma in chronic ulcers that are atypical or refractory to standard wound care. Early biopsy, adequate immunohistochemical evaluation, and systemic staging are essential for diagnosis. Recognition of the angiocentric variant is particularly important because its vascular destruction may mislead clinicians toward infectious, vasculitic, or neutrophilic diagnoses. In this patient, timely histopathologic confirmation allowed appropriate radiotherapy and an excellent clinical response.

## Conclusions

Primary cutaneous CD30-positive anaplastic large cell lymphoma is an uncommon entity that should be considered in the differential diagnosis of chronic, non-healing ulcers, particularly in elderly patients without clear infectious or inflammatory etiology. This case highlights the diagnostic challenge posed by atypical clinical presentations and rare histopathologic variants, such as the angiocentric subtype, which may mimic vasculitic or infectious processes due to its vascular involvement and tissue destruction.

Accurate diagnosis requires a comprehensive approach integrating clinical findings, histopathology, and immunohistochemistry, with demonstration of strong CD30 expression and exclusion of systemic disease. Early biopsy is essential in lesions that do not respond to conventional management, as delayed diagnosis may contribute to prolonged ineffective treatment and potential progression of disease burden. In this patient, localized disease responded favorably to electron beam radiotherapy, consistent with previously reported outcomes in the literature. Recognition of this entity and its variants is critical to ensure timely diagnosis, guide staging, and optimize therapeutic outcomes.
